# Sexual Harassment in the Workplace: Consequences and Perceived Self-Efficacy in Women and Men Witnesses and Non-Witnesses

**DOI:** 10.3390/bs12090326

**Published:** 2022-09-08

**Authors:** Daniela Acquadro Maran, Antonella Varetto, Cristina Civilotti

**Affiliations:** 1 Department of Psychology, Università di Torino, 10124 Torino, Italy; 2 Clinical Psychology Unit, Città della Scienze e della Salute, Corso Bramante 88, 10126 Torino, Italy

**Keywords:** sexual harassment, workplace, consequences, self-efficacy

## Abstract

Despite the numerous advances made in Italy over the years in the study of sexual harassment in the workplace (SHW), research has focused exclusively on victims, perpetrators, and their relationships, and not on the consequences that the experience of sexual harassment can produce in witnesses. The present study aims to address this gap by examining how the indirect experience of SHW, in conjunction with variables such as gender, age, self-efficacy, and coping strategies, affects the mental health status of witnesses of SHW. A sample of 724 employees completed a questionnaire that included a modified version of the Sexual Experience Questionnaire (SEQ), the Oldenburg Burnout Inventory (OLBI), the General Health Questionnaire (GHQ), the Satisfaction With Life Scale (SWLS), and the Emotional Self-Efficacy Scale (RESE). Of the group, 321 participants reported witnessing sexual harassment in the workplace (28.2% of women and 16.2% of men). Results show that witnesses were younger than participants who described themselves as non-witnesses. Results also show that women and men who were witnesses were more likely to suffer the emotional and psychological consequences of the experience than non-witnesses. In addition, female witnesses expressed more positive emotions than men, which enabled them to manage their anxiety and emotional states when triggered in response to sexual harassment in the workplace. Finally, a significant association was found between perceptions of mental health and age, gender, experience with SHW, and self-efficacy strategies. The findings underscore the importance of sexual harassment intervention in the workplace, women and men who witness sexual harassment suffer vicarious experiences, psychological impact, exhaustion, disengagement, and negative feelings.

## 1. Introduction

Sexual harassment in the workplace (hereafter SHW) has been officially recognized since the 1970s as a form of violence to be prevented, and several studies have been conducted on it since then (see, e.g., [1,2]). Fitzgerald et al. [3] define this phenomenon as unsolicited and unwanted sexual behavior that is perceived by the victim as humiliating, offensive, and disabling in terms of their own safety and psychophysical well-being. The International Labor Organization (ILO) describes SHW as a series of repeated, unsolicited, non-reciprocal, and fully imposed harassments by the perpetrator that can have serious undesirable effects on the person [4]. SHW may include acts such as groping, intrusive looks, comments, and/or jokes about the victim’s body/clothing/uterus, use of sexually explicit language or innuendo about the victim’s private life, comments about sexual orientation, or even sexual/erotic contact and viewing of pornographic audio/video material. Chappell and Di Martino [5] provide the same definition in their study and also point out that perpetrators often hold more prestigious positions or have more power in the workplace than victims. For this reason, victims may be afraid to fight back or file formal complaints.

Direct experiences of SHW can be very disabling for both the individual and the organization. Research has shown that bullying can threaten physical, psychological, and occupational well-being [6]. In a summary of studies conducted by the European Commission in Northern European countries, it was found that in 7 of the 75 studies reviewed, more than half of the respondents suffered from negative consequences on general health and well-being [7]. The effects reported by victims included psychosomatic symptoms such as muscle pain and problems of a physical and psychological nature. The most recurrent emotions are anxiety, anger, stress, humiliation, loss of confidence, personal and professional dissatisfaction, and, above all, a deterioration in interpersonal relationships, especially with colleagues. As far as physical symptoms are concerned, those affected mainly report gastrointestinal problems, headaches, insomnia, nausea, loss of appetite, and weight loss [8]. As for mental health, the most serious problems are depressive disorders and post-traumatic stress disorder [9]. The suffering of people in relation to work also leads to deterioration from an organizational point of view. Phenomena such as absenteeism, turnover intentions, and job dissatisfaction can affect organizational performance [10]. Individuals also often experience deterioration in their work performance [11]. Organizational culture also suffers, SHW creates a stressful environment in which victims experience important effects such as loss of trust, confidence, and sense of justice toward the organization and its leadership, a reality in which workers ultimately conclude that they count for nothing to the organization [12].

### 1.1. Consequences of SHW in Witnesses

SHW has been discussed for decades in the scientific literature and in sociopolitical organizations, and there are numerous studies addressing this aspect to guide experimental research, dissemination, and prevention campaigns in the face of increasing and broader awareness by organizations and stakeholders. Unfortunately, the impact of SHW affects not only the direct victims, but also the witnesses of SHW who live in a climate characterized by these dysfunctional behaviors. As early as the late 1990s, Fitzgerald and her colleagues analyzed the potential consequences of SHW, emphasizing that perceptions of such phenomena can lead to deterioration in the physical health of both direct and indirect victims [2,13,14]. These studies suggest that perceptions of gender discrimination, sexual harassment, and other forms of organizational mistreatment can affect women’s and men’s well-being, even if they are not directly affected by SHW.

Some gender differences have been identified in research. Kobrynowicz and Brans-combe [15] indicated that men’s perceptions of SHW are associated with high levels of assertiveness and low self-esteem. Richman et al. [16] found that men’s and women’s perceptions of SHW resulted in diametrically opposite psychological states. In men, SHW was associated with worsening mental health. Schmitt et al. [17] examined the possible consequences of this perception and found that it was both physically and psychologically harmful for women, whereas it had no significant effects for men. One possible explanation suggested by the authors is that women are more likely to be victims of SHW than in other areas. This would lead to more attention being paid to this phenomenon. The study by Harnois and Bastos [18] investigated the phenomenon of SHW and its consequences in men and women. The results showed that the perception of SHW in women was associated with negative effects on the psycho-physical health of the participants. This supports the concept that the perception of SHW can be theorized as a social stressor [19]. Perceptions of the presence of SHW were positively associated with negative effects on physical and emotional well-being in both genders. In line with Siuta and Bergman [20] and Hansen, Garde, and Persson [21], it seems appropriate to refer to experiences of sexual harassment as stressors, also in light of the definition of Kahn and Byosiere [22], who define work stressors as stimuli generated at work that have negative physical or psychological consequences for a significant proportion of individuals exposed to them [23]. These stimuli may characterize a work environment that can be understood as discretionary, in which the stimuli are transmitted differently from individual to individual, or they may permeate the entire work group and thus be potentially available to all members of the group. According to the authors, this also applies to the phenomenon of SHW, which can act either directly at the individual level on the victim—as a discretionary stimulus—or indirectly at the group level on the members—as an environmental stimulus—which would have similar negative effects. Also, in the study presented by Bowling and Beehr [24], workplace bullying is clearly negatively associated with victim well-being, supporting the hypothesis that bullying is a workplace stressor that has effects similar to other workplace stressors such as SHW. Takaki, Taniguchi, and Hirokawa [25] examined the association between SHW and physical consequences, many of which were found to be significant. The authors analyzed data from questionnaires sent to employees (N = 1642) of 35 health care facilities in Japan. The results suggest that stress responses due to SHW could affect health through direct biological effects, prolonged physiological activation, and lack of repair or by affecting lifestyle and health-related behaviors. As suggested by Mathews et al. [26], exposure to these types of stressors could lead to burnout. In their study, 38% of 129 participants reported experiencing at least one SHW episode in their careers.

### 1.2. SHW and Perceived Self-Efficacy

Self-efficacy is a construct introduced by Bandura [27] that represents one of the core mechanisms of personal agency. Self-efficacy is a person’s belief that he or she is capable of organizing and performing the actions necessary to cope with future situations. It is an expression of a person’s self-regulatory abilities and influences the way he or she regulates his or her behavior, thoughts, and affect, as well as the decisions he or she makes and the efforts and persistence he or she undertakes [28,29,30]. According to Bandura, people can successfully achieve their goals in difficult situations if they believe they can perform the required actions [29]. Overall, self-efficacy has been shown to protect against negative psychological factors such as stress and burnout [31]. In general, higher levels of self-efficacy have been shown to positively impact various workplace outcomes by influencing the way individuals interpret their environment. Self-efficacy has been associated with more effective coping with workplace stressors, leading to greater job satisfaction and lower intention to quit [32]. According to Bandura [29], individuals with high self-efficacy are more able to cope with workplace stressors and therefore less likely to avoid frustrating situations by quitting. Self-efficacy appears to have five main effects on behavior. It influences the choices an individual makes based on belief in success or failure; it mobilizes the individual to try harder to succeed; it provides perseverance in the face of obstacles and negative outcomes; it facilitates thought patterns that tell the individual he or she can accomplish the task; and it reduces stress and depression associated with fear of future failure [33]. Self-efficacy appears to play a central role in SHW; research has found that witnesses with high levels of self-efficacy were more likely to actively help or defend their peers, whereas witnesses with lower levels of self-efficacy were more likely to be passive [34,35]. In the study by Hellemans et al. [36], witnesses with low self-efficacy had a greater fear of intervening. This finding is important because it shows the influence of a witness’s personal resources on his or her (non)intervention in the context of SHW.

### 1.3. Current Study

In Italy, the National Institute of Statistics [37] estimates that 8,816,000 women (43.6% of the population) between the ages of 14 and 65 have been sexually harassed in some way during their lifetime, and that 3,118,000 women (15.4%) have been victims of sexual harassment in the last three years. Looking only at the types of sexual harassment also found in the 2008–2009 survey, the estimate of women sexually harassed in the three years prior to the survey increased from 3,778,000 (18.7%) in 2008–2009 to 2,578,000 (12.8%) in 2015–2016. For the first time, sexual harassment was also found among men; an estimated 3,754,000 men were harassed in their lifetime (18.8%), 1,274,000 in the last three years (6.4%). The severity of the harassment suffered varies greatly by gender, with 76.4% of women considering it very or fairly bad, compared to 47.2% of men. In addition, an estimated 1,404,000 (8.9%) women were victims of SHW; 425,000 (2.7%) in the last three years. The vast majority of victims (69.6%) consider the incident to be very or fairly serious. However, in 80.9% of cases, victims did not talk about it with anyone at work. Failure to report victimization experiences to colleagues and supervisors is due to the fear of being perceived as incompetent, inefficient, or inadequately prepared to deal with behaviors that may be considered part of the work environment [38].

In this context, it is important to note that, in 2021, the European Institute for Gender Equality (EIGE), an autonomous agency at the European level, published a gender equality index for the 28 countries of the European Union, based on six areas (work, money, knowledge, time, power, and health). The report shows that Italy has improved significantly in terms of gender equality, but is still below the European average [39]. Apart from this consideration, and despite the numerous advances made in Italy over the years in the study of the phenomenon of sexual harassment, to our knowledge, research has mainly focused on the victims, the perpetrators, and their relationships (e.g., [40,41,42]), and not on the consequences that the experience of sexual harassment can cause in the witnesses. The present study aims to fill this gap in the Italian scientific landscape. The aim of this study was to analyze the consequences of SHW episodes in self-defined witnesses and the perceived self-efficacy that could influence the intention to intervene [34,35,36]. To better understand the experience of being a witness and the role of gender, a comparison was made between male and female witnesses and non-witnesses.

The literature suggests that the consequences are the result of a specific stressor. Therefore, perceived mental health, life satisfaction, and burnout were analyzed, as has been done in other studies around the world with primary victims of SHW (e.g., [43,44,45]). In addition, to assess attitudes toward the intervention, self-efficacy was assessed in terms of the ability to express negative and positive feelings related to SHW episodes. In this context, behaviors characteristic of the experience of SHW were assessed to measure consequences and attitudes toward the intervention.

The overall goal of the study was to examine how the experience of SHW, in conjunction with variables such as gender, age, and coping strategies, affects witnesses’ mental health. To better describe the phenomenon, the following hypotheses were also formulated based on the literature review described below, such as gender differences.

(1) Women who witnessed SHW were more likely to suffer the emotional and psychological consequences of the experience than men and female non-witnesses.
(2) Women who witnessed SHW had more difficulty managing their stress than men and female non-witnesses.
(3) Women who witnessed SHW were more inclined to express negative emotions and less inclined to express positive emotions than men and female non-witnesses.

## 2. Materials and Methods

Participants were asked to anonymously complete a self-administered questionnaire. The first part described the purpose of the questionnaire and included instructions for answering it (including the contact details of the authors of this paper for any doubts or problems), as well as the informed consent form and the declaration of anonymity and privacy. In addition, following the study of Fitzgerald et al. [46], the following description of SHW was given, “Sexual harassment was defined as any unwelcome sexual conduct or other form of discrimination based on sex that violates the dignity of men and women in the learning and working environment, including physical, verbal, or nonverbal conduct. Examples of sexual harassment include (a) implicit or explicit solicitation of offensive or unwanted sexual services; (b) display of pornographic material in the workplace, including in electronic form; (c) use of sexist criteria in any type of interpersonal relationship; (d) implicit or explicit promises of facilities and privileges or professional advancement in return for sexual services; (e) threats or retaliation for refusing sexual services; (f) unwanted and inappropriate physical contact; (g) verbal comments about the body or comments about sexuality or sexual orientation that are perceived as offensive”. The second part of the questionnaire included a request to indicate whether participants had ever witnessed SHW (response = yes/no). The third part of the questionnaire included scales on emotional and psychological consequences, perception of the phenomenon, and coping with the suffering. The last part of the questionnaire included sociodemographic data (e.g., gender, age).

To assess the experiences of witnesses of SHW, the Sexual Experience Questionnaire was used (SEQ, [3]). SEQ is the most widely used and validated measure of sexual harassment [47,48] and asks participants to indicate, on a scale of 1 (never) to 5 (often), how often they have been the target of sexually harassing behavior within the past year. Examples used in this survey include “During the past 12 months, have you been in a situation where any of your supervisors or coworkers … Made sexist remarks to you”. Higher scores indicate more SH victimization. For the purposes of this study, the third-person questions were reformulated in third person: “During the past 12 months, have you been in a situation where any of your supervisors or co-workers … Made sexist remarks to your colleague or other employee or client…”. This scale was only considered for participants who answered “yes” to the question of whether they witnessed SHW. In this study, items from SEQ were aggregated (see [23,48]) (Cronbach’s alpha = 0.94).

The Oldenburg Burnout Inventory (OLBI; [49]) is an instrument for assessing burnout and work engagement. It contains both positively (e.g., “I find my work a positive challenge” or “After work, I have enough energy for my leisure activities”) and negatively (e.g., “During my work, I often feel emotionally drained” or “Over time, one can become disconnected from this type of work”) worded items. This allows the two main dimensions of burnout to be measured; exhaustion, as the result of excessive physical, emotional, and cognitive effort associated with the long-term consequences of the particular demands of a given job, and disengagement (from work, understood as turning away from it in general, from the object of the work, and from its content). These aspects concern the relationship between workers and their work, especially identification with the job and willingness to stay in the same job. The instrument consists of 16 items with a Likert scale ranging from “strongly disagree” to “strongly agree.” (Cronbach’s alpha = 0.85).

The General Health Questionnaire, 12-item version (GHQ-12; [50]; Italian version by Picardi et al. [51]), as described by Shevlin and Adamson [52], belongs to a family of questionnaires for respondents’ self-assessment of psychiatric disorders in community and clinical contexts, as well as for the assessment of disorders of normal functioning and the presence of stress symptoms. The original version consists of 60 items, whereas the version presented in the present study is a follow-up version consisting of exactly 12 items. The items are asked in the form of questions (e.g., “In the past two weeks, have you felt able to concentrate on what you are doing?”) and include a response scale with three response options (from as usual to much less than usual) (Cronbach alpha = 0.81).

The Satisfaction with Life Scale (SWLS; [53]) was used to assess satisfaction with one’s life in general in relation to a general cognitive process. The instrument consists of five statements about specific general aspects of life (e.g., “The conditions of my life are excellent”), which were rated on a Likert scale from 1 = strongly disagree to 7 = strongly agree. (Cronbach’s alpha = 0.87).

The Regulatory Emotional Self-Efficacy Scale (RESE; [54]) is an instrument designed to assess perceived self-efficacy in coping with negative affect and expressing positive affect. The theoretical basis of this instrument lies in the concept that self-efficacy beliefs are dynamic rather than static factors that can be enhanced by coping experiences as a result of the individual’s ability to self-reflect and learn from experiences [29]. In terms of self-efficacy in dealing with positive and negative emotions, the authors refer to the belief that one is able to cope with stress and emotional states (e.g., joy, anger) when they are triggered in response to adverse events. This self-assessment scale includes 12 items (e.g., “Express joy when something good things happen to you?” or “Avoid getting upset when others give you a hard time?”), which are assessed in two subscales: POS (4 items) and NEG (8 items). The NEG subscale also consists of the anger–irritation (ANG 4, items) and dejection–stress (DES, 4 items) subscales (Cronbach alpha = 0.86).

For the scales for which no Italian version was available, they were translated from British English and then back-translated [55]. The translation was done by the authors and two research assistants to agree on a final version.

### 2.1. Procedure

The research project was approved by the Ethics Committee of the University of Turin (Prot. N. 456048/2018). The organizations were contacted with a request for a questionnaire about SHW. The criterion for inclusion was that they were public and private labor organizations in Northern Italy. The exclusion criterion was whether they were voluntary associations or non-profit foundations. A letter of invitation was sent to the heads of the organizations with which we were in contact based on previous work. We asked them to provide us names of people they had already been in contact with. A month after the contacts began, we sent out about thirty letters of invitation. Seven organizations responded positively to the invitation. The other organizations declined or did not respond for various reasons (e.g., lack of time for the project or organizational changes). The organizations that expressed interest received a detailed explanation of the research project. Along with the questionnaires, several ballot boxes were delivered to all sites where employees could have kept their completed questionnaires—given the heterogeneous distribution of employees, the ballot boxes were placed primarily at the organizations’ headquarters, two for each floor and a single ballot box for the other sites. The employees were informed about the research topic, the modalities of voluntary and anonymous participation, and the corresponding deadlines for placing the questionnaires in the corresponding ballot boxes. All participants were informed that their participation was voluntary, that they could leave the interview at any time, and that their responses would remain anonymous. In addition, participants were informed that they could avoid answering if the question worried them, and that if they had negative feelings, they could contact free services offering psychological support. The study was conducted in accordance with Italian privacy regulations. Two weeks were initially allocated for the completion of the questionnaires, which were then extended by a further ten days until the final collection of the questionnaires (information about the schedule and the research topic was also clearly highlighted on the ballots themselves to avoid any ambiguity).

### 2.2. Participants

The questionnaire was distributed in seven different organizations, five of which were private (four companies involved in the production and/or management of goods and services for users and one from the social care sector) and two public (one from the administrative sector and the second from the public health sector). It should be noted that some of the participating organizations were easily identified by the participants of the research due to the number of employees and the type of activity. Therefore, to ensure the anonymity of the participants and the participating organizations, the activities of the organizations were categorized as public/private without providing further information. The estimated total number of potential participants in the study is approximately 1500 individuals, of which 733 employees completed the questionnaires and 724 were considered valid (nine participants did not answer the gender question).

The majority of participants worked in a company with more than 200 employees (37.4%), 21.7% had between 16 and 50 employees, 20.2% between 1 and 15, 12.6% between 51 and 100, and 6.4% in a company with 101 to 200 employees. The majority of participants were employed in a private organization (58.2%), with the remainder employed in a public organization. Overall, 58.4% of the sample were women, 59.1% were single, 36.3% were married, and 4.3% were separated/divorced. Two participants were widowed. Participants were on average 38.75 years old (range 19–65, SD = 13.13). They had work experience ranging from a few months to 44 years (M = 17.41, SD = 12.83), 58.1% had a permanent employment contract, and 44.7% had a college degree.

### 2.3. Statistical Analysis

The data were processed with SPSS version 28 (IBM Corp., Armonk, NY, USA). To assess the significance of differences between witnesses and non-witnesses, χ^2^ tests were used. The Cramer’s V value was calculated to estimate the effect size. As a post hoc test, standardized Pearson residuals (SPRs) were calculated for each cell to determine which cell differences contributed to the results of the χ^2^ test. SPRs whose absolute values were greater than 1.96 indicated that the number of cases in that cell was significantly greater than expected (in terms of over-representation) if the null hypothesis was true, with a significance level of 0.05 [56]. The data were also analyzed using t-test to examine the experience of SHW in witnesses. ANOVA to measure differences between women and men witnesses and non-witnesses. Eta squared was calculated to estimate the effect size. Differences were considered statistically significant when *p* < 0.05. Finally, a multiple regression analysis was used to understand whether perceived mental health can be predicted based on gender, age, SHW, and self-efficacy.

## 3. Results

A total of 321 participants reported being witnesses to SHW (28.2% women and 16.2% men). Among non-witnesses, 30.2% were women and 25.4% were men (see Table 1). On average, female witnesses to SHW were 37.17 years old (range 19–65, SD = 13.21), male witnesses were 36.78 years old (range 20–62, SD = 11.70), while women non-witnesses of SHW were 41.42 years old (range 19–65, SD = 13.34) and men non-witnesses were 38.57 years old (range 21–65, SD = 13.22) (F = 6.87, *p* = 0.002, η2 = 0.092). Regarding years of work experience, female witnesses of SHW had 16.90 years of work experience (range 1–40, SD = 12.63), male witnesses had 17.10 years (range 1–43, SD = 12.39), while female non-witnesses of SHW were 19.14 years old (range 0–41, SD = 12.54) and male non-witnesses were 16.07 years old (range 0–44, SD = 13.50) (F = 1.21, *p* = 0.170, η2 = 0.089). Regarding SHW experience, women reported more dysfunctional behaviors than men (M = 26.33, SD = 9.47 and M = 24.47, SD = 11.00, respectively; t = 2.27, *p* = 0.024, Cohen’s d = 0.176).

As shown in Table 1, single men and married/cohabiting woman are the two categories that report significantly fewer SHW experiences. Women working in the public sector and in organizations with 51 to 100 and 101 to 200 employees, respectively, are more likely to witness SHW, while men in the public sector and in organizations with more than 200 employees report more dysfunctional behaviors.

In Table 2, there is the distribution of response in women and men witnesses and non-witnesses of SHW. Findings indicated that men witnesses were more prone than others to express disengagement, negative feelings such as anger, and dejection–stress. Women witnesses were more prone than others to express positive feelings.

Correlation analysis showed that when participants (women and men) witnessed SHW, life satisfaction decreased (r = −0.12, *p* = 0.029). Finally, multiple regression was performed to predict perceived mental health based on gender, age, SHW, and self-efficacy. Linearity was assessed using partial regression plots and a plot of student residuals against predicted values. Independence of the residuals was assessed with a Durbin–Watson value of 1.922. Homoscedasticity was assessed by visual inspection of a plot of student-specific residuals against the non-standardized predicted values, and there was no evidence of multilinearity assessed by tolerance values greater than 0.1. The normality assumption was met, as determined from a Q–Q plot. The multiple regression model statistically significantly predicted perceived mental health, F(6, 690) = 5.266, *p* < 0.001, adj. R^2^ = 0.13, albeit with a modest effect size. All six variables contributed statistically significantly to prediction, *p* < 0.05. Regression coefficients and standard errors are found in Table 3.

## 4. Discussion

Overall, the results of this study show that perceptions of mental health were significantly predicted by the variables of age, sex, exposure to SHW, and self-efficacy strategies. The effect size was modest because some of the complexity of the phenomenon-which includes psychological, group, organizational, and social aspects was likely not fully accounted for in the modeling. Nonetheless, this is a very important finding because it shows how the phenomenon of SHW affects not only the direct victim but also those who experience it indirectly. This finding is consistent with previous recent studies that, albeit using different methodologies, show that SHW is one of the risk factors at all levels of investigation, from the psychological impact on the individual to the consequences for organizational climate and the welfare parameters of society as a whole [57,58,59].

Witnesses to SHW were younger than participants who identified as non-witnesses. While Powell [60] found that age did not affect how women perceived sexual harassment, Reilly, Lott, and Gallogly [61] found that younger individuals were more likely to tolerate sexual harassment than older individuals. Ford and Donis [62] found that younger women were least likely to tolerate sexual harassment, while younger men were most likely to tolerate sexual harassment. The authors found that tolerance of sexual harassment increases with age in women up to age 50, but decreases thereafter. For men, however, they found the opposite age effect, i.e., tolerance of sexual harassment decreased up to age 50, but acceptance increased thereafter. Foulis and McCabe [63] also found that age did not correlate with Australian workers’ perceptions of sexual harassment. In our study, the results confirmed Padavic and Orcutt’s [64] study that younger workers take the phenomenon of sexual harassment more seriously than older workers (see also [65]).

Our results also confirm the Hypothesis 1: women and men who witnessed sexual harassment were more likely to suffer the emotional and psychological consequences of the experience than non-witnesses, confirming the Hypothesis 1 of this study. However, male witnesses suffered more than women by distancing themselves and expressing negative emotions such as anger and dejection–stress. These results did not confirm Hypothesis 2 (which stated that women who witnessed SHW had more difficulty managing their stress than men and female non-witnesses) and are consistent with Richman–Hirsch and Glomb [66]. Nevertheless, this result is very interesting. Traditionally, studies have focused on female victims of SHW, sociodemographic characteristics, organizational and male-dominance culture, consequences, etc. [5] Fewer studies have been conducted with men, focusing on analysis of their experiences and consequences as witnesses of SHW. The results of the Fourth European Working Conditions Survey, based on 30,000 face-to-face interviews with workers in 31 European countries, show that 2% of all workers are exposed to sexual harassment at work [67]. This means that colleagues, supervisors, and others have contributed to the misconduct. According to Hansen, Garde, and Persson [21], while SH can be understood as a unique discretionary stimulus when experienced directly by a target, it can also manifest as an environmental stimulus that permeates the work context and becomes something that everyone is exposed to in their environment. As mentioned earlier, SHW can lead to a generally stressful work environment that affects employees other than those directly affected by the misconduct [23]. Raver and Gelfand [68] also showed that the effects of SHW extend to group-level outcomes by demonstrating the detrimental effects on team conflict and cohesion. In addition, Berdahl, Magley, and Waldo [69] found that while both genders believe that sexual coercion, unwanted sexual attention, and lewd comments are a form of SHW, men also clearly indicate that punishment for deviating from the masculine gender role (i.e., being harassed as “not masculine enough” [70]) is sexually harassing [38]. Studies show that the men most at risk are those who do not appear sufficiently masculine [14]. Thus, even when men feel anger when they perceive that a member of their own group (and thus potentially themselves) is being harassed, they do not intervene (e.g., [6]). This non-intervention seems to be related to the need to maintain a sense of identification with the gender group; the cost to self might be perceived as a risk [71]. Otherwise, the result could be a sense of powerlessness, driven by the need to intervene to protect the members of the group and their identification with the group. Over time, these feelings can cause suffering, with consequences such as psychological discomfort, exhaustion, and burnout [72].

In addition, women who witnessed SHW expressed more positive emotions than men, which enabled them to manage their anxiety and emotional states when triggered in response to SHW events. Thus, Hypothesis 3, which stated that women who witnessed SHW were more inclined to express negative emotions and less inclined to express positive emotions than men and female non-witnesses, could not be confirmed. This result may be related to the findings of the study by Veletsianos et al. [73]. The authors found that women use different coping strategies to deal with harassment. One of these is resistance, a term we have used to describe women’s refusal to accept harassment or to remain silent or passive. Resistance is a reactive coping strategy, and strategies in this domain included persistent attempts to talk, persistence in general, asserting one’s voice and authority, turning to the community, and using self-protective measures. As Hashmi et al. [74] point out, thanks to the #MeToo campaign, SHW problems and their coping strategies are increasingly seen as structural problems and not just individual-level problems. The witnesses in our study may have been exposed to the “New Deal” for SHW, which influenced how they dealt with the phenomenon [75]. In 2016, prior to the #MeToo momentum, Johnson et al. [76] surveyed 250 professional women in the US about the prevalence of SHW and the impact on their work; they also interviewed 31 women in the US about their individual experiences. After #MeToo, they conducted a second survey of 263 women in September 2018 and reconnected with some of the previously surveyed women to find out if they had noticed any changes or changed their views. The results show the benefits of #MeToo in reducing sexual harassment over two years; women said the movement helped them realize they were not alone in their experiences.

### 4.1. Implications and Application Scenarios of the Study

The results of this study demonstrate the importance of intervening in SHW episodes. Women and men who witness suffer from their vicarious experiences, negative mental health, exhaustion, alienation, and negative feelings. Preventive measures and interventions are needed in the organization. Changing the organizational climate and context that fosters SHW is critical to reducing the phenomenon. Establishing clear zero-tolerance policies and procedures is part of changing the normative environment that fosters SHW. Organizations that proactively develop, disseminate, and enforce policies and procedures on violence against women have the lowest incident rates [77]. In addition, programs that promote witness intervention are important for reducing SHW [78]. Witnesses can potentially confront and stop harassers, report incidents, and support victims [79,80]. Many victims respond passively because they perceive the risk of reporting the incident to be too high; they may rely on others to act on their behalf [81]. By communicating norms that address harassment, witnesses could play a role in changing the group, organizational, and cultural context that supports SHW [82]. Identifying PWD is not enough to motivate intervention; witnesses must take responsibility for their actions [79]. However, multiple witnesses may lead witnesses to assume that their help is not needed and make them feel less responsible (diffusion of responsibility [83]). Witnesses may also attribute responsibility for their intervention to the victim’s colleagues or other members of the group [84]. It might be useful to promote values characteristic of both genders to activate responsibility for intervening. For men, this responsibility could be consistent with masculine roles such as honor and protection [85]. For women, it might be consistent with self-protection and resistance as individual and collective strategies for coping with an environment that might tolerate SHW. Companies could help witnesses stop workplace misconduct. For example, training could be provided to address lack of confidence in one’s own abilities by focusing on specific behaviors that witnesses can use to effectively intervene. Bowes-Sperry and O’Leary-Kelley [79] offered a typology of behaviors that might be useful for such training. The typology classifies possible witness actions along two dimensions, immediacy (immediate action vs. subsequent action) and involvement (direct involvement vs. indirect involvement). For example, episodes with high immediacy and involvement require the witness to take an active and recognizable action, such as asking the harasser to stop. In contrast, behaviors with low immediacy and involvement occur when bystanders later support the victim, for example, by privately encouraging the victim to report the incident. Training could take into account the phenomenon of audience inhibition, which is the concern witnesses have about what others will think of them if they act [83]. Male witnesses, for example, might believe that their intervention (to protect the victim or prevent the perpetrator) will result in a loss of social status if norms of loyalty to members of their own group stand in the way of intervention. Increasing empathy and the importance of personal norms that support intervention may override perceived social norms that contribute to audience inhibition. When an intervention requires that an aggressive member of one’s group be stopped, witnesses may be persuaded to intervene by portraying the actions of aggressors in one’s group as violating group norms and damaging the group’s reputation [84,85,86,87]. Finally, as suggested by Lee et al. [72], it is also important to include in a training program the opportunity to break down stereotypes and myths about SHW to increase the likelihood that witnesses will intervene in high-risk situations. Further research could examine the effectiveness of including witness training in SHW prevention programs. Studies could compare the effectiveness of training for witnesses and non-witnesses with SHW. This could contribute to a better understanding of readiness to intervene and what types of programs increase that readiness.

### 4.2. Limitations of the Study and Future Research Directions

As far as we know, this is the first study conducted in Italy on the phenomenon of SHW in relation to witnesses and non-witnesses. The strength of the project lies in its innovative character, but it is important to consider some limitations that hopefully can be overcome in future studies. First, this was a cross-sectional study. An adequate, but non-random, sample was used for this study. We recognize that the participants in this study may not represent the general population of Italian workers. Willingness to participate in a survey about SHW may be influenced by organizational policies regarding the phenomenon, organizational climate, and previously adopted prevention and intervention strategies. For organizations, the decision to promote or not to promote this survey could imply a particular sensitivity to the phenomenon. A further study could analyze the relationship between the organization’s prevention strategy and the perception of the phenomenon by the organization’s employees. In addition, there could be a bias in participation. Participants might tend to answer a questionnaire in a way that conveys a positive image of themselves or of the organization they belong to (socially desirable responding; [88]). This could mean that participants did not identify themselves as victims and perpetrators; they could describe the phenomenon as witnesses but with greater involvement. Further research could consider the combined use of questionnaires and interviews to better understand the phenomenon and its meaning in an organizational context. Another limitation is that we included participants from different organizations. Therefore, it was not possible to identify specific patterns or episodes of SHW. It might be useful to examine an episode in a particular context using a different method. For example, the mixed method could be useful to describe SHW from different perspectives [89]. In addition, we did not consider the possible relationship between the victim and the perpetrator, their gender, and their sexual orientation. Therefore, further research needs to consider factors such as the perceived severity of the experience, the impact of multiple minority statuses and intersectional oppression on SHW [20], and the organizational values and norms that promote workplace misconduct. Because the nature of the relationship and gender are important predictors of intervention intent [90], it may be interesting to analyze perceptions of the phenomenon in relation to gender in the victim–offender dyad. Future research could use the vignette method to analyze how gender and the nature of the victim–offender relationship influences the intention to intervene in SHW. Finally, it is important to anchor this study in the specific Italian sociocultural context, which may differ from that of other countries [39]. Therefore, this study may not be transferable to other sociocultural contexts.

## 5. Conclusions

In summary, this study has shown that in addressing the serious problem of sexual harassment in the workplace, attention must be focused not only on the direct victims, but also on those who witness it, because they themselves may develop forms of discomfort and because sexual harassment contributes to creating a negative climate for the individual and for the organization itself. Although this is a cross-sectional study without randomization, it clearly shows the need for timely and appropriate intervention in the sociocultural context in which the organization is anchored. In the Italian context, for example, phenomena such as sexism, gender stereotypes, and a tolerance of sexual harassment that is not accepted in other countries still seem to be present [39]. If nothing is done in this regard, either preventively or to curb the phenomenon, there is a risk that harassment will continue in a self-reinforcing cycle. In terms of change and active transformation, it seems crucial to sensitize the widest possible audience of men and women and to promote knowledge and awareness of the problems of hostile and benevolent sexism, homophobia, patriarchal views, and gender stereotypes that still exist in our society. Therefore, it is important and essential that the principles of gender equality and respect for others are taught in all workplaces through appropriate and timely training, prevention, and monitoring.

## Figures and Tables

**Table 1:** Characteristic of the participants (N = 724). Values expressed in column percentage.

	Witnesses		Non-Witnesses		χ^2^	*p*	*V*
	Women (n = 204)	Men (n = 117)	Women (n = 219)	Men (n = 184)			
Marital Status:					12.70	0.002	0.20
- Single	60.0	65.7	47.8	67.8 *			
- Married/Cohabiting	33.8	30.6	47.3 *	29.9			
- Separated/Divorced	6.2	3.7	4.9	2.3			
Educational degree:					4.97	0.547	0.06
- Middle school	4.9	8.5	9.2	9.7			
- High school	49.3	50.8	46.3	44.1			
- University	45.8	40.7	44.5	46.2			
Type of organization:					31.42	0.001	0.21
- Public	44.1 *	34.5	54.8	28.6			
- Private	55.9	65.5	45.2	71.4 *			
Organization size:					30.68	0.002	0.12
- <15 employees	23	18.6	17.7	23.3			
- 16–50 employees	24	21.2	24.7	18.3			
- 51–100 employees	18.5 *	9.3	13.5	8.3			
- 101–200 employees	9.5 *	4.2	6	4.6			
- >200 employees	25	46.6 *	38.1	44.4			
Type of contract:					7.85	0.049	0.10
- permanent work	50.5	57.1	63	61.3			
- temporary work	49.5	42.9	37	38.7			

Note. χ^2^ = Chi-square value; *p* = *p* value; *V* = Cramer’s V value; * = Cells with overrepresentation of subjects.

**Table 2:** Perceived mental health, life satisfaction, burnout, and self-efficacy; comparison between witnesses and non-witnesses of SHW (one-way ANOVA) (N = 724).

	Witnesses		Non-Witnesses		*F*	*p*	*η*2
	Women (n = 204)	Men (n = 117)	Women (n = 219)	Men (n = 184)			
OLBI-Exhaustion	21.99 (12.50)	21.55 (3.05)	21.43 (2.88)	21.38 (2.41)	0.45	0.717	0.043
OLBI-Disengagement	20.50 (12.30)	23.56 (23.68)	19.65 (7.32)	19.22 (2.26)	3.41	0.017	0.062
GHQ-12	22.30 (4.16)	21.60 (9.40)	21.36 (3.99)	20.88 (3.06)	2.59	0.052	0.044
SWLS	22.37 (6.31)	22.88 (5.63)	23.31 (5.91)	23.71 (5.87)	1.79	0.148	0.031
RESE-POS	16.00 (7.55)	15.03 (3.11)	15.76 (3.53)	14.70 (3.47)	2.89	0.035	0.031
RESE-ANG	11.23 (3.15)	12.69 (9.27)	11.26 (3.15)	12.00 (2.97)	3.36	0.019	0.035
RESE-DES	11.66 (3.04)	13.28 (3.57)	12.03 (3.46)	12.93 (2.96)	9.05	0.001	0.042

Note. F = Fischer’s value; *p* = *p* value; η2 = Eta squared.

**Table 3:** Multiple regression results for perceived mental health.

GHQ	B	95% CI for B		SE B	β	R^2^	ΔR^2^
		LL	UL				
Model						0.14	0.13 **
Constant	26.486	24.10	28.87	1.21			
Age	−0.47	−1.25	−0.32	0.40	−0.04		
Gender	−0.10	−0.04	−0.02	0.02	−0.02		
SEQ	0.10	0.03	0.05	0.02	0.01		
RESE-POS	−0.12	−0.16	−0.01	0.04	−0.08		
RESE-ANG	−0.23	−0.36	−0.10	0.07	−0.15		
RESE-DES	−0.36	−0.12	−0.05	0.04	−0.33		

Note. Model = “Enter” method in SPSS statistics; B = unstandardized regression coefficient; CI = confidence interval; LL = lower limit; UL = upper limit; SE B = standard error of the coefficient; β = standardized coefficients; R^2^ = coefficient of determination; Δ R^2^ = adjusted R^2^. ** *p* < 0.01. The gender variable is calculated as female vs. male.

## Data Availability

The datasets generated for this study are available on request to the corresponding author.
